# Positive, negative, neutral—or unknown? The perceived valence of emotions expressed by young autistic children in a novel context suited to autism

**DOI:** 10.1177/13623613211068221

**Published:** 2022-02-16

**Authors:** Claudine Jacques, Valérie Courchesne, Suzanne Mineau, Michelle Dawson, Laurent Mottron

**Affiliations:** 1 Université du Québec en Outaouais, Canada; 2 CIUSSS du Nord-de-l’Île-de-Montréal, Canada; 3 Université de Montréal, Canada

**Keywords:** autism, context, facial emotion, interests, valence, young children

## Abstract

Starting early in life, autistics are characterized as having atypical facial expressions, as well as decreased positive and increased negative affect. The literature on autistic facial expressions remains small, however, with disparate methods and results suggesting limited understanding of common autistic emotions. Furthermore, unlike non-autistics’ emotions, autistics’ emotions have been assessed without considering this population’s characteristics. In this study, the valence of young children’s facial expressions was thus rated as positive, negative, neutral, or “unknown”—a term for perceived emotions observers do not understand. Facial expressions were assessed using the Montreal Stimulating Play Situation, a context incorporating potential autistic interests. Comparing 37 autistic and 39 typical young (27–56 months) age-matched children, we found no group differences in expressed positive, negative, and neutral emotions. We did find differences in unknown emotions, which were unique to the autistic group. Preliminary data also showed that autistic children’s repetitive behaviors co-occurred with positive, neutral, and unknown emotions, but not with negative emotions. In a novel context that considers their characteristics, we did not find decreased positive or increased negative emotions in young autistic children. Instead, they uniquely expressed emotions perceived as unknown, showing the need to improve our understanding of their full emotional repertoire.

**Lay abstract:**

Autistic people are believed to have emotions that are too negative and not positive enough, starting early in life. Their facial expressions are also persistently judged to be unusual, as reflected in criteria used to identify autism. But it is possible that common autistic facial expressions are poorly understood by observers, as suggested by a range of findings from research. Another issue is that autistic emotions have always been assessed in contexts suited to non-autistics. In our study, the facial expressions of young autistic and typical children were rated as positive, negative, neutral, or “unknown”—a category we created for emotions that observers notice but do not understand. These emotions were assessed using a context suited to autistic children, including objects of potential interest to them. We found that in this context, autistic and typical children did not differ in positive, negative, or neutral facial emotions. They did differ in unknown emotions, which were found only in autistic children. We also found that repetitive behaviors in autistic children co-occurred with positive, neutral, and unknown emotions, but not with negative emotions. In a context which suits their characteristics, autistic children do not show emotions that are too negative or not positive enough. They do show emotions perceived as unknown, which means we need to improve our understanding of their full emotional repertoire.

## Introduction

Atypical emotional expression has been included within formal autism diagnostic criteria since their outset. DSM-III’s brief criteria alluded to “bizarre” or “lack of” responses (APA, 1980). DSM-III-R specified no or “markedly abnormal” facial expression, as well as failure to smile or “a fixed stare” (APA, 1987), while DSM-IV-TR had “marked impairment” in use of facial expression (APA, 2000). Current DSM-5 criteria include a lack of facial expressions (APA, 2013). Atypical emotional expression also features in items from major autism diagnostic and screening instruments (Autism Diagnostic Interview-Revised-ADI-R, Kim et al., 2013; Autism Diagnostic Observation Schedule-ADOS, Gotham et al., 2007; Modified Checklist for Autism Toddlers-revised-M-CHAT, Robins et al., 2014; Social Communication Questionnaire-SCQ, Chesnut et al., 2017) in the form of deficits in facial expressions (e.g. inappropriate, extreme, unvarying, limited), in sharing enjoyment, and in responsive smiling.

Nonetheless, autism research has been dominated by investigations of how autistics process the facial expressions of typical individuals (Lozier et al., 2014; Trevisan & Birmingham, 2016; Uljarevic & Hamilton, 2013). The literature on facial expressions in autism is, in comparison, small: in a systematic review and meta-analysis spanning 1967–2017, Trevisan et al. (2018) found a total of 37 articles. This literature of small studies (autistic *N*s range: 4–54) encompasses a wide range of participants (mean age 2–44 years), facial expression measurement methods, types of expressions, and outcomes. Main results of combined effects include that autistic groups, versus various comparison groups, showed less frequent and “less socially-congruous” (i.e. less suited to the social context; p. 1587) facial expressions. The quality of autistic expressions was characterized as “much more awkward, odd, unusual, mechanical, or otherwise irregular in appearance” (Trevisan et al., 2018, p. 1596). Effects were greater in autistics classified as intellectually disabled versus those not, and in studies of younger versus older participants. However, even within the 10 studies of autistic children with a mean age under 6 years, all judged intellectually disabled (7 studies) or their intellectual functioning unknown (3 studies), effects varied widely.

More recently, Grossard et al. (2020) used a two-dimensional scale of emotion “recognizability” and “credibility” and characterized verbally instructed and imitated (from an avatar) facial expressions in school-aged autistic children as more ambiguous and less socially meaningful. Bangerter et al. (2020) used automated facial expression encoding and classified the expressions of 124 autistics aged 6–54 years who watched funny videos as either “under-responsive” (*N* = 89) or “over-responsive” (*N* = 35). In a systematic review, Briot et al. (2021) summarized the use of such automated methods to assess differences in various facial metrics (e.g. symmetry, complexity, synchrony, ambiguity) potentially underlying typical observers’ judgments of autistic facial emotions as “odd, stilted, or mechanical” or “strange.”

Studies on facial expressions in young autistic children exist within the context of a broader literature investigating early development in autism (e.g. home video, infant sibling, and/or parent report studies) and combining facial with other forms of expression to assess emotions. Findings in this literature have contributed to proposals that positive emotions are reduced and negative emotions are increased in autistic children starting early in development (Clifford et al., 2013; Garon et al., 2009, 2016; Macari et al., 2017; Paterson et al., 2019; Zwaigenbaum et al., 2013). In this direction, Hirschler-Guttenberg et al. (2015) considered facial expressions in conjunction with other indications (e.g. vocal and body expressions) and reported reduced positive emotions in preschool autistic children within “emotion regulation” paradigms (see also Cibralic et al., 2019; Mazefsky et al., 2013). However, Macari et al. (2018) tested autistic, typical, and developmentally delayed toddlers using standardized strategies (not involving “social interaction or persons displaying emotions”) for inducing positive (joy) and negative (anger, fear) emotions. They found no support for proposals that autism is characterized by more negative and/or less positive emotions. Instead, they found “a complex and surprising emotional landscape” (Macari et al., 2018, p. 832) in young autistic children.

Indeed, across development, autistic children have been singled out for the extent to which they express facial emotions that observers do not understand. For example, Yirmiya et al. (1989) reported that young autistic children had “ambiguous” facial expressions “not displayed by any of the other children.” Loveland et al. (1994) reported that facial expressions they judged to be “bizarre” and “unrecognizable” were common among autistic individuals. The ensuing literature (Briot et al., 2021; Trevisan et al., 2018; Zane et al., 2019 for reviews) has further characterized autistic facial expressions as incongruous, unclear, inappropriate, flat, odd, awkward, disorganized, stilted, strange, and/or unnatural. This suggests an important role for autistic emotional expressions which typical observers perceive as such, but have difficulty interpreting or cannot interpret at all.

It is also plausible that issues of interpretation exist at a more fundamental level than the categorization of facial expressions into traditional basic emotions, such as happiness, sadness, anger, disgust, fear, and surprise. Indeed, the nature, meaning, usefulness, universality, and number of basic facial expression categories in the general population are increasingly being questioned (Barrett et al., 2019; Brooks et al., 2019; Du et al., 2014; Hassin et al., 2013). The quality of valence, from positive to neutral to negative, is more fundamental and underlies emotional experience and expression (Barrett, 2006; Shuman et al., 2013), including facial expressions (Gendron et al., 2018; Kring & Sloan, 2007). Typical observers’ poor understanding of some autistic facial expressions may exist at the level of valence, such that they may perceive an emotion is being expressed, but cannot interpret it as positive, negative, or neutral. For observers, these perceived emotions are, therefore, “unknown.” Using the term “unknown” would shed needed light on the aforementioned array of terms applied to autistics expressing emotions, and would be a first step toward acknowledging and quantifying the extent to which common autistic facial expressions are poorly understood.

There are also issues of context. To date, autistic facial expressions have been assessed using contexts suited to the characteristics of typical or non-autistic individuals. Assessments in children have used spontaneous or instructed responses to stimuli featuring typical models and/or situations suitable for typical children (Capps et al., 1993; Grossman et al., 2013; Loveland et al., 1994; Macdonald et al., 1989; Rozga et al., 2013); typical social interaction situations (Capps et al., 1993; Dawson et al., 1990; Filliter et al., 2015; Kasari et al., 1990; Reddy et al., 2002; Snow et al., 1987; Yirmiya et al., 1989); prompts suited to typical children (Volker et al., 2009); videos that typical individuals find funny (Bangerter et al., 2020); and/or sets of objects that are interesting to non-autistic children (Bieberich & Morgan, 2004; Filliter et al., 2015; Hirschler-Guttenberg et al., 2015; Yirmiya et al., 1989).

Conversely, autistic children have had their facial expressions of emotion assessed without considering which contexts potentially suit this population’s interests, aptitudes, and characteristics. For example, Kaale et al. (2018) found that 45% of autistic children aged 24–60 months showed no positive affect (facial or other emotional expressions) during typical joint engagement in a context featuring a “standard set of toys” (ball, car, stuffed animal, picture book; blocks, marbles, toy phones, miniature figures). Autistic children’s low frequency of positive affect in such typical joint engagement contexts contrasts with the high frequency in typical children (Kasari et al., 1990). Autistic children may, however, show relatively frequent positive affect not directed toward another person (Snow et al., 1987), with potential consequences for how people they interact with, including parents, perceive their emotions. Furthermore, while both increased negative and decreased positive emotions were reported in autistic youth by their parents, neither was found in the autistic offspring’s self-reports (Samson, Hardan, et al., 2015; Samson, Wells, et al., 2015). These results question the use of a single proxy or parent-report measure to assess emotions in younger autistic children (overview in Paterson et al., 2019) and suggest the importance of both interpretation and context in improving our understanding of emotional expressions in autism.

We thus investigated the valence of facial emotions, including the possibility of “unknown” emotions, expressed by young autistic children within the Montreal Stimulating Play Situation (MSPS), a context developed to consider their interests, aptitudes, and characteristic (Jacques et al., 2018). MSPS incorporates objects of potential interest to autistic children, who are free to explore these objects in play periods with three different levels of structure (free, semi-free, and semi-structured play), and in the absence of any effort to suppress atypical autistic behaviors. It is thus possible to compare multiple aspects of object exploration and repetitive behaviors in autistic versus typical young children. Using MSPS, we previously found that young autistic children, compared to age-matched typical children, showed increased overall and specific (hand flapping, close gaze at objects, arm movements) repetitive behaviors, but no decrease in object exploration, in frequency, duration, and different objects explored (Jacques et al., 2018).

In this study, our primary aim was to assess whether the valence of expressed facial emotions during MSPS differed between the autistic and typical groups. We hypothesized that in a context suited to young autistic children, autistic and typical children would be similar in prevalence of expressed positive and negative facial emotions. Our explicit use of an “unknown” emotion category is novel in the autism literature but, based on what the terms and results detailed above consistently imply, we hypothesized that unknown emotions would be found primarily in autistic children. We therefore compared the prevalence (as duration, frequency, and proportion of children) of expressed positive, negative, neutral, and unknown facial emotions in autistic and age-matched typical young children, both for the entire MSPS and within the different play period structures.

Our secondary and exploratory aim was to assess, in the autistic children, the co-occurrence of expressed facial emotions with characteristically autistic repetitive behaviors. Whether repetitive behaviors in autism are positive or negative experiences remains in question (King, 2019), and there is uncertainty as to whether these behaviors reflect pleasure or distress or neither (Harrop et al., 2014; Mottron, 2017; Reddihough et al., 2020). Despite this uncertainty, these behaviors are targeted for reduction or elimination by a variety of early autism interventions (Boyd et al., 2011; Grahame et al., 2015; Lovaas, 2003; Rogers & Dawson, 2010; see French & Kennedy, 2018 and Rodgers et al., 2020 for systematic reviews). This lends importance to asking whether specific targeted characteristically autistic repetitive behaviors, such as hand-flapping (Akers et al., 2020), co-occur with expressed emotions in autistic children. Therefore, in the entire MSPS, we documented the co-occurrence of expressed positive, negative, neutral, and unknown facial emotions with the three repetitive behaviors (hand flapping, close gaze at objects, arm movements) found in the study by Jacques et al. (2018) to be increased in autistic children. Because this aspect of the study was exploratory, we did not have hypotheses.

## Methods

### Participants

Participants in this study were a subset of the 92 children (49 autistic, 43 typical, aged 20–69 months) from the study by Jacques et al. (2018) who were assessed with MSPS. The current study required MSPS videos to provide a sufficiently clear view of each child’s face for accurate coding of facial expressions. As explained below, only children whose MSPS videos were of sufficient quality for facial expression coding were included in this study. This resulted in a final sample of 37 autistic and 39 typical children, aged from 27 to 56 months.

All children were recruited from the greater Montreal area: autistic children from the Banque de données et de participants Autisme-HSMRDP, and typical children from local day-care centers. The autistic children were diagnosed by a multidisciplinary team composed of a psychiatrist and at least one other professional (psychoeducator or psychologist). All autistic children scored above autism spectrum cut-offs on the Autism Diagnostic Observation Schedule (Generic or second edition) and met DSM-5 autism diagnostic criteria, while none had clinical indications of an identifiable genetic condition. Typical children’s parents completed an in-house screening questionnaire to exclude the presence of autism, developmental delays, or behavioral issues (questionnaire missing for 3 out of 39 typical children).

Autistic and typical groups were matched on age and sex. Mullen Scales of Early Learning (MSEL) composite and domain scores were significantly lower in the autistic children, as expected (Courchesne et al., 2019). See Table 1 for participant characteristics (age, sex, ethnicity; MSEL) and Supplementary Table 1 for ADOS scores. The study was approved by HRDP and Université du Québec en Outaouais research ethics committees. Written informed consent was obtained from each child’s parent.

**Table 1.** Participant characteristics.

Total sample	Autistic	Typical	*p*
	*N* = 37	*N* = 39	
Mean age in months (*SD*)	45.8 (10.5)	41.1 (14.1)	0.124
Boys: girls	27:10	29:10	0.387
White	30 (81.1%)	34 (87.2%)	
Black	2 (5.4%)	5 (12.8%)	
Asian	2 (5.4%)		
Latinx	3 (8.1%)		
Sample with available MSEL scores, mean (*SD*)	*N* = 30	*N* = 36	
MSEL composite	74.0 (27.69)	103.0 (24.7)	< 0.001
MSEL visual reception	38.78 (19.10)	52.71 (15.47)	0.002
MSEL fine motor	33.19 (17.42)	50.29 (15.18)	< 0.001
MSEL receptive language	34.34 (17.48)	50.66 (14.86)	< 0.001
MSEL expressive language	33.41 (18.99)	50.67 (16.73)	< 0.001

MSEL: Mullen Scales of Early Learning.

Age and MSEL: T-tests. Boys: girls: chi-square. MSEL composite are standard scores (mean 100, *SD* 15). MSEL visual reception, fine motor, receptive language, and expressive language are all T-scores (mean 50, *SD* 10).

### Montreal Stimulating Play Situation

#### Overview

MSPS is a protocol developed primarily for assessing object exploration and repetitive behavior in young autistic children. However, it can also be used to assess additional observable behaviors. A key element of MSPS is its incorporation of objects which possess perceptual or informational properties of interest for an autistic population (e.g. books with written texts, magnetic letters and numbers; Mottron, Dawson, & Soulières, 2013; Ostrolenk et al., 2017). As reported in the study by Jacques et al. (2018), object selection for MSPS was informed by the existing literature and a survey of autism professionals, followed by changes after pilot testing and testing in a first sample of typical and autistic children. Other key elements of MSPS are that children are free to explore objects of their choice as they wish across different types of play (free, semi-free, semi-structured) and that there is no effort to reduce or redirect atypical autistic behaviors.

MSPS was administered by one of two psychoeducators with an expertise in autism, who was in the testing room with the child, while the child’s caregiver observed MSPS from behind a one-way mirror. A trained cameraman recorded the child’s behaviors on video, and two trained coders naïve to child diagnosis rated the videos. The coders were recruited from universities (non-graduate studies), had only incidental knowledge of autism (i.e. as expected in the general population, with no additional knowledge or experience), and had no information about the objectives of the study. For a full description of MSPS and how it was developed and administered, see Jacques et al. (2018), and for images of the MSPS testing room, see Supplementary Figure 1.

#### Objects

MSPS was revised in the course of its development, as described in Jacques et al. (2018), such that children were assessed with two similar versions (MSPS-A, MSPS-B) with minor differences in number and type of objects. In this study, MSPS-A, with 34 objects, was administered to 21 autistic and 24 typical children, while MSPS-B, with 40 objects, was administered to 16 autistic and 15 typical children. At the start of MSPS, room layout was the same for all children (apart from minor differences across versions), including the location of objects, most of which were distributed throughout the room, 11 of which were hidden in a box. See Supplementary Table 2 for MSPS-A and MSPS-B object lists.

#### Administration and play periods

MSPS administration takes approximately 30 min divided into four play periods, always in the same order: free play 1 (5 min), semi-free play (5 min), semi-structured play (no set time limit), and free play 2 (5 min). Both free play periods allowed children to explore their choice of objects and move freely in the testing room. In the semi-free play period, the children could also explore their choice of objects and move freely, but when the child explored an object, the psychoeducator activated it or copied the child’s actions. In the semi-structured play period, the 11 objects previously hidden in the box were presented by the psychoeducator to the child one at a time for a maximum of 2 min or three repeated activations per object. In this period, children also had free access to all the other MSPS objects (those originally displayed in the room, and those from the box already presented to the child). In the free play 2 period, the child had free access to all MSPS objects, including the 11 from the box.

Unless there was a risk of injury, children’s behaviors were not stopped or redirected. However, free play 1 and semi-free play were interrupted if the child was inactive for more than 2 min (*n* = 1 typical child). Also, after semi-structured play, one typical child indicated he wanted to go back to his parents and did not complete free play 2. Despite this, and despite semi-structured play not having a set time limit, duration of all play periods did not differ across groups. See Supplementary Table 3 for play period durations and group comparison statistics.

### Recording and video quality

The entire session was recorded for each child. However, as explained by Jacques et al. (2018), the trained cameraman who recorded MSPS was in the room for MSPS-A (21 autistic and 24 typical children), but not in MSPS-B (28 autistic and 19 typical children), where he was outside the room and used two remote-controlled cameras. Although the child’s face was clearly visible most of the time, the resolution in the first MSPS-B videos recorded was insufficient for emotion coding to be possible. This was determined by two authors (CJ and VC) in conjunction with the two coders, and technical improvements were made afterward to allow for a better resolution. Children (12 autistic, or 43%; 4 typical, or 21%) whose MSPS-B videos were determined to be of insufficient quality for facial expression coding were thus not included in this study. Using Fisher’s exact test, groups did not significantly differ in excluded versus included MSPS-B videos, *p* = 0.096.

Children whose videos were excluded also did not differ in age (autistic children 48.3 months, *SD* = 9.78; typical children 44.5 months, *SD* = 16.66, *p* = 0.576); however, an autistic child aged 69 months and a typical child aged 20 months were among those who had videos excluded.

### Video coding

#### Emotions

MSPS involves a long duration (~30 min per child) of observations in a natural setting. Several methods have been used to measure facial expressions in autism (Trevisan et al., 2018), with no consensus regarding how the expressions of young autistic children should be assessed in this kind of context. Algorithms can be used to automatically code facial actions, but this is distinct from identifying the valence of facial emotions as perceived by human observers (Martinez, 2019), thus our choice to use ratings coded by trained typical observers. Furthermore, while valence is considered dimensional and to vary in intensity of expression (Kring & Sloan, 2007), we aimed to continuously assess the frequency and duration of expressed facial emotion valence regardless of intensity for the entire MSPS.

We thus developed a novel simple rating grid based on facial expressions of emotions with positive, negative, neutral, and unknown valence. Each valence was associated with a simple operational description. Positive and negative emotions are straightforwardly defined, with neutral emotions entailing an absence of valence (not positive or negative; no expression; Kring & Sloan, 2007), and unknown emotions were described as: “Impossible to identify the facial expression as positive, negative, or neutral.” The raters used this code when unable to identify the facial expression of emotion as positive, negative, or neutral, while nevertheless perceiving that an emotion was being expressed. A fifth code, impossible to determine, was added for instances when scoring was not possible (e.g. hidden face). See Table 2 for descriptions of each coded valence of expressed emotions. The default valence was set to neutral and the coders were instructed to change this code to either positive, negative, unknown or impossible to determine according to the definitions provided, thus all codes have a beginning and an end from which a duration could be extracted. We are aware that this passage between two emotions can be considered as a midpoint, which does not represent a specific emotion (see Young et al., 1997).

**Table 2.** Valence of facial emotions with descriptions.

Valence of facial emotions	Descriptions
Positive emotions	Smile without teeth showing (e.g. closed mouth) Smile with teeth showing
Negative emotions	Open mouth (fear) Frowning Lips pulled down Crying
Neutral emotions	No facial expression
Unknown emotions	Impossible to identify the facial expression as positive, negative, or neutral
Impossible to determine	Impossible to see the facial expression (cannot see the face) Incorrectly positioned to see the face

All facial emotions expressed by children during the entire MSPS were entered in the coding system (Observer XT 11) by the two trained coders, who were naïve to group status and the study’s purpose, and as noted above re MSPS coders, had only incidental (i.e. general-population level) knowledge of autism. The coders were trained by one of the authors (CJ or VC) until they reached an inter-rater reliability of 90% across all codes, which means that at least 90% of the time, coders were putting the same code “on” at the same time and putting it “off” to put the same other code “on” also at the same time; discrepancies between the two coders were discussed with CJ and VC until consensus was reached. For the study, 20% of included videos were randomly selected to calculate inter-rater reliability across all the codes. Given that frequency and duration of expressed facial emotions were analyzed separately, we calculated inter-rater reliability separately for the two variables, using intraclass correlation coefficients (ICC) estimate (two-way mixed-model absolute agreement single measure; Shrout & Fleiss, 1979). ICC was 0.915 (range from 0.803 to 0.980; good to excellent reliability), *p* < 0.001 for frequency (seeing the same facial emotion during the same time period; facial emotion overlapping) and 0.843 (range from 0.713 to 0.957; moderate to excellent reliability), *p* < 0.005 for duration (onset/offset identification of a facial emotion within the same 3 s window by the two coders). See Supplementary Table 4 for the ICC of each individual code.

#### Repetitive behaviors

An autism repetitive behaviors repertoire was used to code MSPS videos. The autism repetitive behaviors repertoire was developed based on the literature and on a survey conducted with clinician experts in autism and was successfully used in Jacques et al. (2018). The final repertoire includes 48 repetitive behaviors, which were computer coded (Observer XT 11) by two trained coders (see information above about MSPS coders). In Jacques et al. (2018), three of the 48 repetitive behaviors were significantly more prevalent (in frequency, duration, and/or proportion of children) in the autistic compared to the typical group; hand flapping, close gaze at objects, and arm movements. In this study, we therefore explored the co-occurrence of these three repetitive behaviors with expressed facial emotions (see Supplementary Table 5, for operational definitions used to code these three behaviors, and Supplementary Table 6 for descriptive data for these three behaviors). A co-occurrence was recorded when an expressed facial emotion and one of the three specified repetitive behaviors were observed at the same time, that is as long as there was overlap, regardless of the duration of this overlap. The procedure for training coders to reliability on the coding of repetitive behaviors was similar to the one used for the coding of emotions (see Jacques et al., 2018, for more details).

### Community involvement statement

This group has a long history of autistics and non-autistics contributing to autism research as equals, in equally diverse roles. Journal policy requires one author (MD) to disclose her diagnosis, because she is autistic. We believe this policy is discriminatory. Another author (SM) is a clinician who works with young autistic children. As detailed in Jacques et al. (2018), many other clinicians were involved in the development of MSPS.

### Statistical analyses

Analyses were performed for the entire MSPS, then for each play period. To assess facial emotions across the different levels of structure within MSPS (free vs semi-free vs semi-structured play), free play 1 and free play 2 were combined to form a free play composite (see Supplementary Table 7a and 7b). All the variables were screened for skewness and kurtosis, which showed that the distribution was not normal considering the variability of frequency and duration data. We also tested the normality of residuals using Shapiro–Wilk tests, which showed that with few exceptions, all for neutral expressed facial emotions (duration in the entire MSPS, duration in each play period), data were not normally distributed (see Supplementary Table 8a to 8d). We therefore opted for the use of non-parametric tests, and Bonferroni corrections were applied to decrease the likelihood of type I errors, leading to an adjusted alpha level of 0.01. Mean ranks for frequency and duration of positive, negative, neutral, and unknown emotional expressions, as well as “impossible to determine” codes, were compared between autistic and typical children using non-parametric Mann–Whitney U tests (Nachar, 2008). Effect sizes were calculated post hoc using Cohen’s *d* for non-parametric tests, both for frequency and duration. Effect sizes were considered small at 0.2, medium at 0.5, and large at 0.8.

Proportion of children in each group who expressed positive, negative, neutral, and unknown emotions, as well as proportion of “impossible to determine” codes, were compared using Fisher’s exact test.

For the autistic group, we assessed co-occurrence of positive, negative, neutral, and unknown expressed facial emotions with the three characteristically autistic repetitive behaviors from Jacques et al., 2018: hand-flapping, arm-movements, and close gaze at objects. This was assessed as proportion of children showing each co-occurrence in the entire MSPS. Statistical analyses were not planned for these exploratory and preliminary data.

## Results

### Valence of expressed facial emotions in the entire MSPS

“Impossible to determine” facial expressions were coded in 100% of children, with no group differences in duration and frequency (duration: *U* = 714.00, *p* = 0.938; frequency: *U* = 717.00, *p* = 0.963; see Supplementary Table 9a and 9b).

Autistic and typical children did not significantly differ in mean ranks for duration and frequency of expressed positive (duration: *U* = 542.50, *p* = 0.063, *d* = 0.437; frequency: *U* = 576.00, *p* = 0.130, *d* = 0.352), negative (duration: *U* = 600.50, *p* = 0.116, *d* = 0.292; frequency: *U* = 592.00, *p* = 0.092, *d* = 0.312), and neutral (duration: *U* = 697.00, *p* = 0.799, *d* = 0.058; frequency: *U* = 691.00, *p* = 0.751, *d* = 0.073) facial emotions. In contrast, expressed unknown emotions were more frequent (*U* = 409.50, *p* < 0.001, *d* = 0.801) and of greater duration (*U* = 409.50, *p* < 0.001, *d* = 0.801) in the autistic group; see Tables 3 and 4. Effect sizes for the non-parametric tests were thus small for duration and frequency of expressed positive and negative emotions, but large for unknown emotions.

**Table 3.** Duration of expressed emotions in autistic and typical children in the entire MSPS, in seconds.

	Autistic (*n* = 37)			Typical (*n* = 39)			*p*	Cohen’s *d*
	*M* (*SD*)	Range	MR	*M* (*SD*)	Range	MR		
Positive	97.75 (95.39)	0–375.15	33.66	142.42 (126.13)	0–548.57	43.09	0.063	0.437
Negative	6.49 (16.32)	0–71.60	41.77	3.43 (14.23)	0–87.85	35.40	0.116	0.292
Neutral	1004.96 (274.73)	364.69–1543.40	39.16	966.58 (278.07)	236.50–1548.76	37.87	0.799	0.058
Unknown	14.11 (36.75)	0–160.95	46.93	0	0	30.50	< 0.001*	0.801

*M*: mean; *SD*: standard deviation; MR: mean rank.

* Significant group difference. Cohen’s *d* is effect size for non-parametric tests.

**Table 4.** Frequency of expressed emotions in autistic and typical children in the entire MSPS, as number of occurrences.

	Autistic (*n* = 37)			Typical (*n* = 39)			*p*	Cohen’s *d*
	*M* (*SD*)	Range	MR	*M* (*SD*)	Range	MR		
Positive	23.16 (21.45)	0–96	34.57	27.92 (19.36)	0–97	42.23	0.130	0.352
Negative	1.30 (3.24)	0–18	42.00	0.46 (1.27)	0–6	35.18	0.092	0.312
Neutral	75.54 (31.81)	17–175	37.68	73.67 (22.87)	11–119	39.28	0.751	0.073
Unknown	3.51 (9.04)	0–39	46.93	0	0	30.50	< 0.001*	0.801

*M*: mean; *SD*: standard deviation; MR: mean rank.

* Significant group difference. Cohen’s *d* is effect size for non-parametric tests.

**Figure 1.**
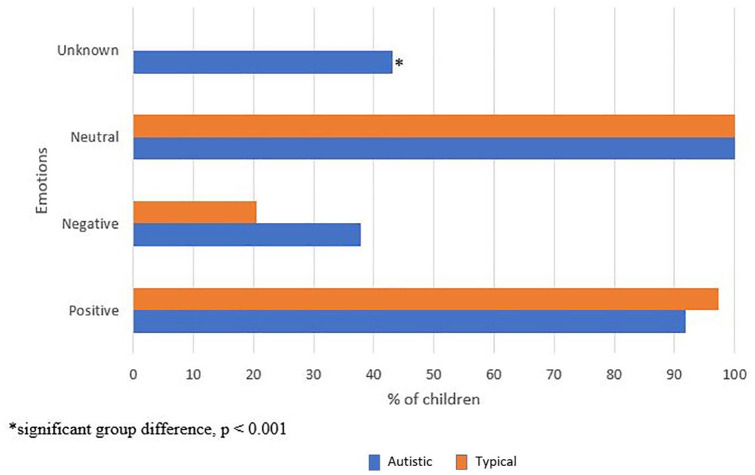
Proportion of autistic and typical children who expressed emotions in the entire MSPS.

There were no significant differences in proportion of autistic and typical children who expressed positive (autistic = 91.9%; typical = 97.4%, *p* = 0.464), negative (autistic = 37.8%; typical = 20.5%, *p* = 0.09), and neutral (autistic = 100%; typical = 100%) facial emotions. Unknown emotions were expressed only by autistic children (autistic = 43.2%, typical = 0%, *p* < 0.001); see Figure 1.

### Valence of expressed facial emotions in each play period

“Impossible to determine” facial expressions were coded in 100% of children in each play period, with no group differences in duration and frequency in free play composite (duration: *U* = 717.00, *p* = 0.963; frequency: *U* = 636.00, *p* = 0.749), semi-free play (duration: *U* = 651.00, *p* = 0.582; frequency: *U* = 656.50, *p* = 0.621), or semi-structured play (duration: *U* = 641.00, *p* = 0.403; frequency: *U* = 685.00, *p* = 0.849; see Supplementary Table 9a and 9b).

No differences were found between groups in frequency and duration for expressed positive, negative, and neutral facial emotions in free play composite (all *p* ⩾ 0.13), semi-free play (all *p* ⩾ 0.16), and semi-structured play (all *p* ⩾ 0.03). Expressed unknown emotions were significantly more frequent (free play composite: *U* = 565.50, *p* = 0.002; semi-free-play: *U* = 585.00, *p* = 0.005; semi-structured play: *U* = 487.50, *p* < 0.001) and of greater duration (free play composite: *U* = 565.00, *p* = 0.002; semi-free play: *U* = 585.00, *p* = 0.005; semi-structured play: *U* = 487.50, *p* < 0.001) in the autistic group during each play period; see Tables 5 and 6. For duration, small effects were found for unknown emotions in free play composite (*d* = 0.379) and semi-free play (*d* = 0.330), while medium effects were found for positive (*d* = 0.533) and unknown (*d* = 0.581) emotions in semi-structured play. For frequency, small effects were found for negative (*d* = 0.205) and unknown (*d* = 0.379) emotions in free play composite; for positive (*d* = 0.312), neutral (*d* = 0.274), and unknown (*d* = 0.330) emotions in semi-free play; and for neutral emotions (*d* = 0.212) in semi-structured play. Medium effects were found for positive (*d* = 0.532) and unknown (*d* = 0.581) emotions in semi-structured play.

**Table 5.** Duration of expressed emotions in autistic and typical children in each MSPS play period structure, in seconds.

	Autistic (*n* = 37)			Typical (*n* = 39)			*p*	Cohen’s *d*
	*M* (*SD*)	Range	MR	*M* (*SD*)	Range	MR		
Free play composite								
Positive	25.11 (32.40)	0–153.76	39.84	24.45 (32.61)	0–124.50	37.23	0.604	0.118
Negative	2.55 (7.68)	0–37.77	40.73	0.55 (2.27)	0–13.01	36.38	0.144	0.198
Neutral	296.99 (127.83)	79.01–558.28	38.49	295.66 (127.47)	72.60–563.47	38.51	0.996	0.001
Unknown	6.93 (22.14)	0–124.89	42.72	0	0	34.50	0.002*	0.379
Semi-free play								
Positive	13.66 (20.66)	0–97.13	38.95	14.15 (19.47)	0–65.44	38.08	0.861	0.039
Negative	0.79 (3.83)	0–29.99	40.05	1.51 (9.45)	0–59.02	37.03	0.164	0.014
Neutral	164.22 (65.41)	39–351.04	42.00	144.18 (56.82)	0–253.21	35.18	0.178	0.137
Unknown	1.85 (6.05)	0–31.31	42.19	0	0	35.00	0.005*	0.330
Semi-structured play								
Positive	58.98 (67.99)	0–277	32.66	103.82 (100.91)	0–461.62	44.04	0.025	0.533
Negative	3.14 (11.26)	0–56.26	39.24	1.36 (4.89)	0–28.83	37.79	0.663	0.064
Neutral	543.75 (180.69)	212.65–883.10	38.89	526.74 (183.14)	118.18–872.54	38.13	0.880	0.033
Unknown	5.34 (18.80)	0–112.56	44.82	0	0	32.50	< 0.001*	0.581

*M*: mean; *SD*: standard deviation; MR: mean rank.

* Significant group difference. Cohen’s *d* is effect size for non-parametric tests.

**Table 6.** Frequency of expressed emotions in autistic and typical children in each MSPS play period structure, as number of occurrences.

	Autistic (*n* = 37)			Typical (*N* = 39)			*p*	Cohen’s *d*
	*M* (*SD*)	Range	MR	*M* (*SD*)	Range	MR		
Free play composite								
Positive	6.59 (6.26)	0–22	40.32	5.74 (6.57)	0–26	36.77	0.479	0.161
Negative	0.59 (1.95)	0–11	40.81	0.08 (0.27)	0–1	36.31	0.130	0.205
Neutral	24.95 (11.68)	7–52	38.62	23.51 (9.54)	5–46	38.38	0.963	0.011
Unknown	1.30 (3.38)	0–15	42.72	0	0	34.50	0.002*	0.379
Semi-free play								
Positive	3.27 (4.19)	0–16	38.66	3.49 (4.23)	0–14	38.35	0.949	0.312
Negative	0.22 (0.85)	0–5	40.08	0.08 (0.48)	0–3	37.00	0.157	0.140
Neutral	13.78 (6.88)	2–37	41.58	11.74 (6.13)	0–28	35.58	0.235	0.274
Unknown	0.73 (2.56)	0–15	42.19	0	0	35.00	0.005*	0.330
Semi-structured play								
Positive	13.30 (13.97)	0–60	32.68	18.69 (13.25)	0–62	43.03	0.025	0.532
Negative	0.49 (1.28)	0–7	39.27	0.31 (0.86)	0–4	37.77	0.651	0.068
Neutral	36.81 (18.65)	9–89	36.11	38.41 (14.71)	2–70	40.77	0.358	0.212
Unknown	1.49 (4.78)	0–28	44.82	0	0	32.50	< 0.001*	0.581

*M*: mean; *SD*: standard deviation; MR: mean rank.

* Significant group difference. Cohen’s *d* is effect size for non-parametric tests.

**Figure 2.**
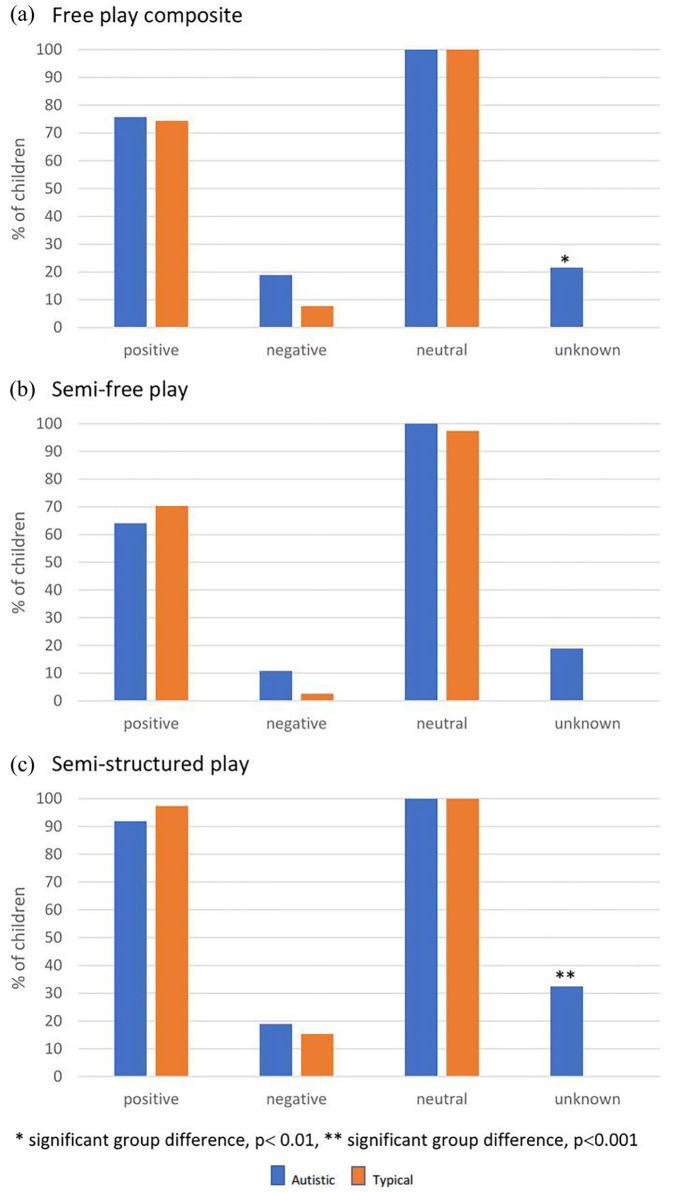
Proportion of autistic and typical children who expressed emotions in each MSPS play period structure.

There were no significant differences in proportion of autistic and typical children who expressed positive facial emotions in free play composite (autistic = 75.7%; typical = 74.4%, *p* = 0.605), semi-free play (autistic = 64.1%; typical = 70.3%, *p* = 0.849), or semi-structured play (autistic = 91.9%; typical = 97.4%, *p* = 0.501); expressed negative facial emotions in free play composite (autistic = 18.9%; typical = 7.7%, *p* = 0.270), semi-free play (autistic = 10.8%; typical = 2.6%, *p* = 0.303), or semi-structured play (autistic = 18.9%; typical = 15.4%, *p* = 0.675); or expressed neutral facial emotions in free play composite (autistic = 100%, typical = 100%), semi-free play (autistic = 100%, typical = 97.4%, *p* = 0.595), and semi-structured play (autistic = 100%; typical = 100%). Unknown facial emotions were expressed only by autistic children: in free play composite (autistic = 21.6%; typical = 0%, *p* < 0.01), semi-free play (autistic = 18.9%; typical = 0%, *p* = 0.013, NS), and semi-structured play (autistic = 32.4%; typical = 0%, *p* < 0.001); see Figure 2.

### Co-occurrence between valence of expressed facial emotions and repetitive behaviors in autistic children

**Figure 3.**
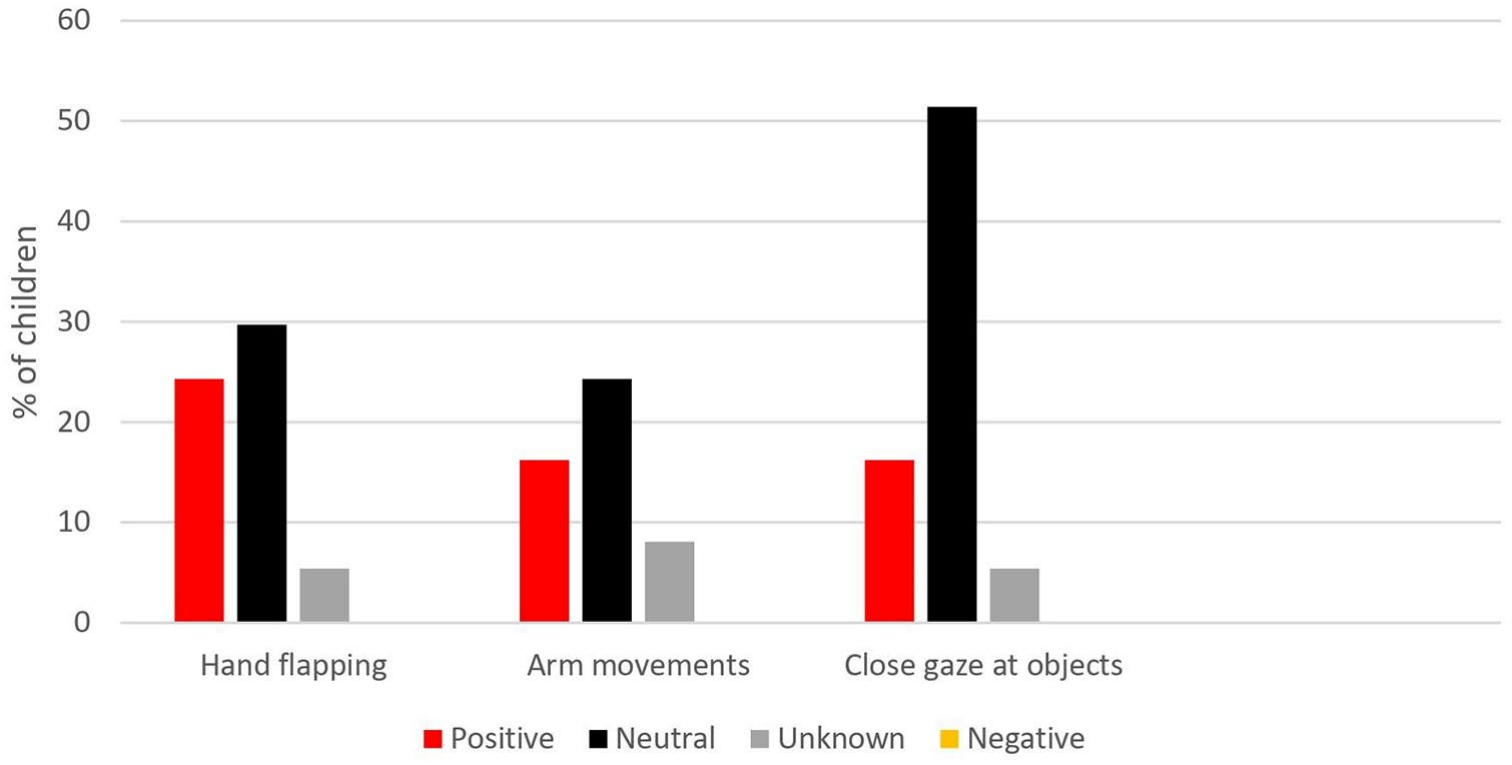
Expressed emotions co-occurring with specific repetitive behaviors in autistic children (as proportion of children showing co-occurrence), in the entire MSPS.

In autistic children, repetitive behaviors co-occurred with expressed positive, neutral, and unknown facial emotions, but not with negative emotions. Proportions of autistic children showing a co-occurrence across the three assessed repetitive behaviors (hand-flapping, arm-movements, and close gaze at objects) ranged from 24.3% to 51.4% for neutral emotions, 16.2% to 24.3% for positive emotions, and 5.4% to 8.1% for unknown emotions. No autistic children showed a co-occurrence between any of the three repetitive behaviors and negative emotions (see Figure 3).

## Discussion

We documented the valence of expressed facial emotions in autistic and age-matched typical young children in a context, MSPS, featuring free play, semi-free play, and semi-structured play periods and incorporating objects of potential interest to autistics (Jacques et al., 2018). In this context, we found no group differences in expressed positive, negative, and neutral facial emotions. We did not find evidence for the decreased positive and/or increased negative emotional expressions, which have been proposed as characteristic of autism starting early in development (Cibralic et al., 2019; Clifford et al., 2013; Garon et al., 2009; Garon et al., 2016; Macari et al., 2017; Mazefsky et al., 2013; Paterson et al., 2019; Zwaigenbaum et al., 2013) though not necessarily found in all contexts (Macari et al., 2018). Indeed, both groups expressed many positive emotions and few negative emotions during MSPS and in each play period.

Previously, we showed that MSPS, a novel situation filled with new information, is a context where young autistic children explore their environment as much as typical children do, thus providing them with similar learning opportunities (Jacques et al., 2018). Current deficit-based autism criteria and theories invoking insistence on sameness, inflexibility (extreme distress in response to changes, difficulties with transitions; APA, 2013), sensory hypersensitivity, sensory dysfunction, and/or sensory overload (Pellicano, 2013; Simmons, 2019; Sinha et al., 2014; Van de Cruys et al., 2014), would predict negative emotions in young autistic children who find themselves in a mostly unstructured unfamiliar context including a large array of unfamiliar objects. In the alternative, strength-based accounts of autism involving enhanced perception and perceptual capacity (Mottron, Bouvet, et al., 2013; Mottron et al., 2009; Remington et al., 2019) suggest that autistics are attracted to information (familiar or not) which they can process well (Hanley et al., 2017), and may benefit from access to more and more complex information of this kind (Nader et al., 2021). This may account for the positive facial emotions expressed by autistic children in MSPS. We believe MSPS could in this way provide insight as to what kinds of contexts may promote autistic development and learning, without autistic children experiencing more negative emotions than their typical peers.

However, we did find group differences in the category of expressed “unknown” facial emotions, that is, facial expressions that were perceived as conveying emotions, but which observers could not interpret and thus could not rate as positive, negative, or neutral. None of the typical children expressed unknown emotions in the entire MSPS, in contrast with 43.2% of autistic children, who also expressed these emotions within each of the different play period structures (range: 18.9% to 32.4%). Our addition and coding of “unknown” expressed emotions is a novel departure from a literature where multiple terms implying a poor and biased understanding (Brewer et al., 2016) of autistic emotional expressions have accumulated, without consensus on how these expressions should be characterized or interpreted (Briot et al., 2021; Grossman et al., 2013; Loveland et al., 1994; Trevisan et al., 2018; Yirmiya et al., 1989). Our approach instead explicitly acknowledges that typical observers may not know how to identify even the valence of certain emotional expressions uniquely shown by autistic individuals. We propose an objective characterization of these “unknown” emotions in autism, which is a first step to better understand their valence starting early in life.

Our findings raise the importance of exploring the extent of unknown autistic emotions in more detail and in multiple modalities of emotional expressions (e.g. tone of voice, verbal expressions, body movements, gestures; Nuske et al., 2013). More avenues for further investigation of unknown autistic emotions may include the use of autistic self-reports in addition to observational measures; replication of this study with autistic observers; and the possibility that a wider array of information, not limited to faces, is used to interpret facial expressions (Aviezer et al., 2008; Hassin et al., 2013). Also, the construction of a catalog detailing unknown expressed emotions (specific descriptions, specific contexts, and so on) could contribute to their better understanding, including whether they indeed convey emotions, as they were perceived and coded in our study, or if some instead are facial movements independent of emotional expression. Working toward more accurate interpretation of facial and other emotions in autism starting early in development carries the possibility of improving on current practices, for example, in how parents and professionals interact with autistics (Sullivan & Lewis, 2003).

Apart from free access to potentially interesting objects and different levels of structure, MSPS as a context features no effort to suppress atypical or repetitive behaviors associated with autism. We were, therefore, able to explore the co-occurrence in autistic children of expressed facial emotions with hand-flapping, arm-movements, and close gaze at objects. If repetitive behaviors are reflecting or are in themselves distressing experiences for autistic people, which remains in question (Baribeau et al., 2020; King, 2019; Mottron, 2017; Reddihough et al., 2020; Samson et al., 2014; Zhou et al., 2021), these behaviors should co-occur with negative rather than positive emotions. Instead, none of the autistic children expressed negative emotions during the entire time they were engaged in any of the three repetitive behaviors. These repetitive behaviors did co-occur with positive emotions in 16.2%–24.3% of autistic children, as well as with neutral emotions in 24.3%–51.4%, and unknown emotions in 5.4%–8.1%. Interpretation of these preliminary and exploratory results is limited by the low occurrence of negative emotions expressed by autistic children in MSPS, as opposed to the high occurrence of positive emotions, which did co-occur with repetitive behaviors (as did the relatively lower-occurring unknown emotions). Our main and preliminary findings together suggest that positive emotions in autism may be increased, and negative emotions decreased, in contexts such as MSPS in which repetitive behaviors are not suppressed. However, comparing expressed emotions in MSPS with those expressed in other contexts will improve our understanding of these latter findings.

This study has several limitations. The sample size limits statistical power. It is possible that with a larger sample, group differences could arise. Non-parametric tests for positive and negative emotions did not show significant differences, and most comparisons showed no or small effects. However, medium effect sizes were found for frequency and duration of positive emotions during the semi-structured play period. Thus, it is possible that if statistical power had been greater, differences could be detected with more positive emotions in typical children during this play period. Future studies with a larger sample will need to explore a possible difference between groups in the expression of positive emotions when children are exposed to a more structured context. It will be important to plan and perform equivalence tests to confirm that there are no differences between groups for positive and negative emotions. Similarities instead of group differences also could arise for unknown emotions. However, the absence of unknown emotions in 39 typical children over the entire course of MSPS suggests that if these are expressed in typical children, they are rare. In the same direction, autistic children’s greater frequency and duration of unknown emotions in the entire MSPS showed large effect sizes, suggesting that this group difference may be robust.

Another limitation is that MSPS was not initially designed to assess facial expressions, and the use of remote-controlled cameras for part of the original sample (28 autistic and 19 typical children) resulted in videos for some children (12 autistic and 4 typical) being excluded due to children’s faces being inadequately visible for coding (see video recording and quality in the “Methods” section, above). In the sample where remote-controlled cameras were used, a greater proportion of autistic (43%) than typical (21%) children’s videos were excluded. Although this was not a significant group difference, it remains possible that the autistic children excluded differed from those included in their facial expressions. In addition, all included videos had emotions coded as “impossible to determine” due to children’s faces not being adequately visible at certain points. However, the duration, frequency, and proportion of children coded with “impossible to determine” emotions did not differ between groups.

Consistent with our purpose, we also used continuous coding with a novel, simple rating grid for expressed facial emotion valence, in a departure from more elaborate and subjective coding systems common in previous studies (Loveland et al., 1994; Grossman et al., 2013). Even so, it is possible that the trained typical raters in this study in addition applied or were influenced by their own emotion recognition abilities and experiences. Consequently, in future studies, using multiple coders could increase the accuracy. Also, emotions were coded without isolating the face area from other available information in the videos, making it possible that this additional information played into how facial expressions were coded as emotions. Furthermore, facial expressions may not consistently reflect human emotions, and while this problem is mitigated at the more fundamental level of valence (Barrett et al., 2019), any such discrepancy may be greater in autism. Nevertheless, facial expressions influence how individuals are perceived, such that atypical expressions in autism affect how autistics are regarded and responded to, from early in life (Filliter et al., 2015) to adulthood (Brewer et al., 2016; Faso et al., 2015).

Furthermore, we used a single context suited to autistics, and in which autistic behaviors are not suppressed, for all children. However, within MSPS, there are play periods with different levels of structure, from free to semi-structured play; our findings suggest there may be group differences in the expression of positive emotions specifically in contexts with more structured play. Accordingly, it would be interesting for future studies to compare frequency and duration of facial emotions across various contexts to assess their impact. Finally, the young autistic children in this study were included regardless of their MSEL scores and were age-matched with typical children whose mean MSEL composite score was two standard deviations higher. That is, compared to age-matched typical peers, young autistic children did not express increased negative or decreased positive facial emotions, despite being assessed as having much lower developmental levels. MSEL scores have been found to be discrepant with autistic children’s higher cognitive potential as revealed by a strength-informed assessment (Courchesne et al., 2019). This, in turn, is consistent with our previous study using MSPS (Jacques et al., 2018), where autistic children’s object exploration suggested interests in information at least as complex and diverse as shown by age-matched children with much higher MSEL scores. This underlines the importance of taking the necessary steps to provide young autistic children with contexts that suit their interests and abilities, which do not suppress their characteristic behaviors, and which appreciate their complex range of emotions (Macari et al., 2018).

## Conclusion

Contexts suited to non-autistics are routinely used to assess all components of autistics’ emotional experience. In this study, we instead used MSPS, a novel context suited to autism, to investigate the perceived valence of autistic facial emotions. Our findings suggest that when young autistic children are free to explore and learn from information they may process well, as in the context of MSPS, their expressed facial emotions are rated as positive as those of age-matched typical children. In another departure from the literature to date, we directly acknowledged that typical observers may not understand and thus cannot interpret commonly expressed autistic facial emotions, even at the fundamental level of valence. By explicitly coding “unknown” as well as positive, negative, and neutral emotions, we found that expressed facial emotions which observers perceived but failed to understand were unique to autistic children, indicating that their full repertoire of emotional expressions remains poorly understood compared to typical children’s. MSPS provides a positive experience to young autistic children, a context which may be used to increase our knowledge of their range of expressed emotions, including emotions that we do not yet understand.

## Supplemental Material

sj-docx-1-aut-10.1177_13623613211068221 – Supplemental material for Positive, negative, neutral—or unknown? The perceived valence of emotions expressed by young autistic children in a novel context suited to autism Click here for additional data file. Supplemental material, sj-docx-1-aut-10.1177_13623613211068221 for Positive, negative, neutral—or unknown? The perceived valence of emotions expressed by young autistic children in a novel context suited to autism by Claudine Jacques, Valérie Courchesne, Suzanne Mineau, Michelle Dawson and Laurent Mottron in Autism

sj-JPG-2-aut-10.1177_13623613211068221 – Supplemental material for Positive, negative, neutral—or unknown? The perceived valence of emotions expressed by young autistic children in a novel context suited to autism Click here for additional data file. Supplemental material, sj-JPG-2-aut-10.1177_13623613211068221 for Positive, negative, neutral—or unknown? The perceived valence of emotions expressed by young autistic children in a novel context suited to autism by Claudine Jacques, Valérie Courchesne, Suzanne Mineau, Michelle Dawson and Laurent Mottron in Autism
