# Supplementary material for: Positive, negative, neutral—or unknown? The perceived valence of emotions expressed by young autistic children in a novel context suited to autism
Source: Autism. 2022 Feb 16;26(7):1833–48. doi: 10.1177/13623613211068221 (PMC9483191; doi:10.1177/13623613211068221)
Supplement: sj-docx-1-aut-10.1177_13623613211068221 – Supplemental material for Positive, negative, neutral—or unknown? The perceived valence of emotions expressed by young autistic children in a novel context suited to autism [file sj-docx-1-aut-10.1177_13623613211068221.docx]

**Supplementary Tables**

Supplementary Table 1.

*Participant characteristics: available ADOS scores for the autistic group*

| **Modules** | **Mean (SD)** |
| --- | --- |
| ADOS G module 1 social communication; N=24  Autism cut-off =12 | 15.71 (3.52) |
| ADOS G module 2 social communication; N=2  Autism cut-off =12 | 16.44 (2.08) |
| ADOS-2 toddler module total social affect + restricted and repetitive behavior; N=4  Autism cut-off=16 (few to no words) or 12 (some words) | 25 (1.73) |
| ADOS-2 module 1 total social affect + restricted and repetitive behavior score; N=1  Autism cut-off=10 (younger than five years) and 9 (five years and older) | 25 |

ADOS-G algorithm autism or autism spectrum cut-off is based on social communication scores. ADOS-2 algorithm autism cut-off is based on social affect and restricted and repetitive behavior. Six ADOS algorithms are missing.

Supplementary Table 2.

*MSPS-A and MSPS-B object lists*

| **MSPS-A and MSPS-B object lists. Objects in bold were in the box at the start of MSPS** | | |
| --- | --- | --- |
|  | MSPS-A | MSPS-B |
|  | n=34 total  **n=11 in box** | n=40 total  **n=11 in box** |
| Abacus | √ | √ |
| Action-reaction game | √ | √ |
| Balls with lights and sounds | √ | √ |
| **Balloons** | **√** | **√** |
| **Big abacus** | **√** | **√** |
| **Boat: hammer and balls** | **√** | **√** |
| Books (3) with written texts | √ | √ |
| Baby bottle |  | √ |
| **Bubble gun** | **√** | **√** |
| Calendar | √ | √ |
| Cylinders with beads | √ | √ |
| Dinosaurs (2) | √ | √ |
| Doll |  | √ |
| **Elephant with throwing balls** | **√** | **√** |
| Empty bucket | √ | √ |
| Frog proprioceptive | √ | √ |
| **Game odors** | **√** |  |
| **Hoops** | **√** | **√** |
| i**-Pad: “The farm 1-2-3” application** |  | **√** |
| **Light and music stars** | **√** | **√** |
| Magnetic letters and numbers | √ | √ |
| Miniature cars (4) | √ | √ |
| Miniature helicopters |  | √ |
| Mirror balls (2) | √ | √ |
| Music box | √ | √ |
| Newspaper | √ | √ |
| Picture dictionary |  | √ |
| Regular dictionary | √ | √ |
| **Remote controlled car** |  | **√** |
| **Remote controlled dinosaur** | **√** |  |
| Slinky | √ | √ |
| Sound blocks | √ | √ |
| **Sound embedding objects** | **√** | **√** |
| Spinning light and sound | √ | √ |
| Stuffed giraffe | √ | √ |
| Tactile balls | √ | √ |
| Telephone sounds | √ | √ |
| Tracks |  | √ |
| Trains (3) |  | √ |
| Vibrating object | √ | √ |
| Visual and sound train | √ | √ |
| **Wheel caterpillar** | **√** | **√** |

Supplementary Table 3.

*Mean duration of each play period for each group*

| Play period | Mean duration in seconds (SD) | | P value |
| --- | --- | --- | --- |
|  | **Autistic** | **Typical** |  |
| Free play 1 | 296.11 (60.93) | 295.83 (41.69) | 0.982 |
| Semi-free play | 288.74 (69.00) | 286.03 (53.19) | 0.855 |
| Semi-structured play | 897.95 (152.00) | 905.46 (123.01) | 0.822 |
| Free play 2 | 305.46 (121.19) | 292.38 (72.79) | 0.587 |

Supplementary Table 4.

*ICCs for each of the 4 coded emotions*

| Emotions | **Frequency** | **Duration** |
| --- | --- | --- |
| **Positive** | .970 | .957 |
| **Negative** | .980 | .940 |
| **Neutral** | .905 | .713 |
| **Unknown** | .803 | .760 |

Supplementary Table 5.

*From the autism repetitive behaviors repertoire, descriptions for the 3 assessed repetitive behaviors in autistic children*

| **Behaviors** | **Description** |
| --- | --- |
| Close gaze at objects | Inspecting object by placing it 3 inches or less from the eyes |
| Hand flapping | Flapping movements of semi-flexed hands and arms up and down and on each side of the body |
| Arm movements | Moving one or both arms (implying the entire arm) |

Supplementary Table 6.

*Duration and frequency for the 3 assessed repetitive behaviors in autistic children*

|  | **Duration (seconds)** | |  | | **Frequency (number of occurrences)** | | | |
| --- | --- | --- | --- | --- | --- | --- | --- | --- |
|  | Total | Range | Mean | SD | Total | Range | Mean | SD |
| **Hand flapping** | 245.58 | 0-62.98 | 6.30 | 14.04 | 89.00 | 0-25 | 2.28 | 5.38 |
| **Arm movements** | 151.91 | 0-64.54 | 3.90 | 12.75 | 56.00 | 0-28 | 1.44 | 4.61 |
| **Close gaze at object** | 771.24 | 0-172.24 | 19.78 | 33.12 | 110.00 | 0-10 | 2.82 | 2.91 |

Supplementary Table 7a.

*Duration of expressed emotions in autistic and typical children in MSPS free play 1 and free play 2, in seconds*

|  | **Autistic (n=37)** | | | **Typical (n=39)** | | |  |
| --- | --- | --- | --- | --- | --- | --- | --- |
|  | *M* (SD) | Range | Mean rank | *M* (SD) | Range | Mean rank | *p* |
| **Free play 1** |  |  |  |  |  |  |  |
| Positive | 12.89 (21.19) | 0-108.13 | 40.27 | 12.70 (22.12) | 0-92.51 | 36.82 | 0.479 |
| Negative | 2.35 (7.65) | 0-37.77 | 40.19 | 0.55 (2.27) | 0-13.01 | 36.90 | 0.247 |
| Neutral | 146.61 (76.07) | 14.92-362.98 | 38.00 | 143.32 (67.84) | 0-270.90 | 38.97 | 0.848 |
| Unknown | 0.95 (3.84) | 0-22.57 | 41.14 | 0 | 0 | 36.00 | 0.018 |
| **Free play 2** |  |  |  |  |  |  |  |
| Positive | 12.21 (25.17) | 0-142.67 | 38.73 | 11.75 (18.66) | 0-80.05 | 38.28 | 0.928 |
| Negative | 0.20 (1.20) | 0-7.27 | 39.03 | 0 | 0 | 38.00 | 0.305 |
| Neutral | 150.39 (87.46) | 0-334.45 | 38.65 | 152.33 (92.87) | 0-372.41 | 38.36 | 0.954 |
| Unknown | 5.98 (21.37) | 0-124.89 | 42.19 | 0 | 0 | 35.00 | 0.005* |

* significant group difference

Supplementary Table 7b.

*Frequency of expressed emotions in autistic and typical children in MSPS free play 1 and free play 2, as number of occurrences*

|  | |  | |  | | | |  | | | | | | | |  | |
| --- | --- | --- | --- | --- | --- | --- | --- | --- | --- | --- | --- | --- | --- | --- | --- | --- | --- |
|  |  | **Autistic (n=37)** | |  | | | | | | | **Typical (n=39)** | | |  | | |  |
|  |  | *M* (SD) | | Range | | | Mean rank | | | | *M* (SD) | | Range | | Mean rank | | *p* |
| **Free Play 1** |  | | |  | |  |  |  |  |  |  |  |  |  |  |  |  |
| Positive | | 3.21 (4.01) | | 0-13 | | | | | 40.47 | | 2.79 (3.93) | | 0-14 | | 36.63 | | 0.430 |
| Negative | | 0.57 (1.95) | | 0-11 | | | | | 40.28 | | 0.08 (0.27) | | 0-1 | | 36.81 | | 0.221 |
| Neutral | | 12.78 (4.52) | | 3-23 | | | | | 39.77 | | 11.92 (5.04) | | 0-22 | | 37.29 | | 0.624 |
| Unknown | | 0.41 (1.67) | | 0-10 | | | | | 41.14 | | 0 | | 0 | | 36.00 | | 0.018 |
| **Free Play 2** |  | |  | |  | | | | |  | |  |  |  |  |  |  |
| Positive | | 3.37 (4.44) | | 0-21 | | | | | 39.49 | | 2.94 (4.32) | | 0-17 | | 37.56 | | 0.696 |
| Negative | | 0.02 (0.16) | | 0-1 | | | | | 39.03 | | 0 | | 0 | | 38.00 | | 0.305 |
| Neutral | | 12.16 (9.78) | | 0-35 | | | | | 37.61 | | 11.59 (7.00) | | 0-26 | | 39.35 | | 0.731 |
| Unknown | | 0.89 (2.35) | | 0-10 | | | | | 42.19 | | 0 | | 0 | | 35.00 | | 0.005* |

* significant group difference

Supplementary Table 8a.

*Normality of data, duration of expressed emotions in autistic and typical children in the entire MSPS, Shapiro-Wilk test*

|  | **Autistic (n=37)** |  | **Typical (n=39)** |  |
| --- | --- | --- | --- | --- |
|  | W | p | W | p |
| Positive | 0.85 | <0.001 | 0.86 | <0.001 |
| Negative | 0.46 | <0.001 | 0.25 | <0.001 |
| Neutral | 0.97 | 0.525 | 0.98 | 0.724 |
| Unknown | 0.44 | <0.001 | - | - |

Note: The test rejects the hypothesis of normality when the p value is less than 0.05.

Supplementary Table 8b.

*Normality of data, frequency of expressed emotions in autistic and typical children in the entire MSPS, Shapiro-Wilk test*

|  | **Autistic (n=37)** |  | **Typical (n=39)** |  |
| --- | --- | --- | --- | --- |
|  | W | p | W | p |
| Positive | 0.85 | <0.001 | 0.92 | 0.009 |
| Negative | 0.45 | <0.001 | 0.42 | <0.001 |
| Neutral | 0.93 | 0.017 | 0.98 | 0.868 |
| Unknown | 0.43 | <0.001 | - | - |

Note: The test rejects the hypothesis of normality when the p value is less than 0.05.

Supplementary Table 8c.

*Normality of data, duration of expressed emotions in autistic and typical children in each play period, Shapiro-Wilk test*

|  | **Autistic (n=37)** |  | **Typical (n=39)** | |  | |
| --- | --- | --- | --- | --- | --- | --- |
|  | W | p | W | | p | |
| **Free play composite** |  |  |  |  | |  |
| Positive | 0.73 | <0.001 | 0.75 | | <0.001 | |
| Negative | 0.39 | <0.001 | 0.27 | | <0.001 | |
| Neutral | 0.98 | 0.626 | 0.98 | | 0.554 | |
| Unknown | 0.36 | <0.001 | - | | - | |
| **Semi-free**  **Play** |  |  |  | |  | |
| Positive | 0.70 | <0.001 | 0.75 | | <0.001 | |
| Negative | 0.21 | <0.001 | 0.15 | | <0.001 | |
| Neutral | 0.97 | 0.497 | 0.97 | | 0.300 | |
| Unknown | 0.35 | <0.001 | - | | - | |
| **Semi-structured play** |  |  |  | |  | |
| Positive | 0.76 | <0.001 | 0.85 | | <0.001 | |
| Negative | 0.31 | <0.001 | 0.31 | | <0.001 | |
| Neutral | 0.96 | 0.232 | 0.98 | | 0.863 | |
| Unknown | 0.30 | <0.001 | - | | - | |

Note: The test rejects the hypothesis of normality when the p value is less than 0.05.

Supplementary Table 8d.

*Normality of data, frequency of expressed emotions in autistic and typical children in each play period, Shapiro-Wilk test*

|  | **Autistic (n=37)** |  | **Typical (n=39)** | |  | |
| --- | --- | --- | --- | --- | --- | --- |
|  | W | p | W | | p | |
| **Free play composite** |  |  |  |  | |  |
| Positive | 0.88 | <0.001 | 0.83 | | <0.001 | |
| Negative | 0.35 | <0.001 | 0.30 | | <0.001 | |
| Neutral | 0.93 | 0.019 | 0.98 | | 0.624 | |
| Unknown | 0.45 | <0.001 | - | | - | |
| **Semi-free**  **Play** |  |  |  | |  | |
| Positive | 0.77 | <0.001 | 0.80 | | <0.001 | |
| Negative | 0.28 | <0.001 | 0.15 | | <0.001 | |
| Neutral | 0.90 | 0.003 | 0.98 | | 0.665 | |
| Unknown | 0.31 | <0.001 | - | | - | |
| **Semi-structured play** |  |  |  | |  | |
| Positive | 0.77 | <0.001 | 0.80 | | <0.001 | |
| Negative | 0.45 | <0.001 | 0.42 | | <0.001 | |
| Neutral | 0.92 | 0.010 | 0.97 | | 0.385 | |
| Unknown | 0.33 | <0.001 | - | | - | |

Note: The test rejects the hypothesis of normality when the p value is less than 0.05.

Supplementary Table 9a.

*Duration of emotions “impossible to determine” in autistic and typical children in the entire MSPS and each play period structure, in seconds*

|  |  | | | |  | | | |  |
| --- | --- | --- | --- | --- | --- | --- | --- | --- | --- |
|  | **Autistic (n=37)** | | | **Typical (n=39)** | | | |  | |
|  | *M* (SD) | Range | Mean rank | *M* (SD) | Range | Mean rank | *p* | |  |
| **Entire MSPS** | 664.95 (258.44) | 200.76-1331.88 | 38.30 | 667.27 (253.76) | 132.57-1368.77 | 38.69 | 0.938 | |  |
| **Free play composite** | 269.99 (139.72) | 77.47-515.57 | 38.38 | 267.55 (124.47) | 44.61-457-04 | 38.62 | 0.963 | |  |
| **Semi-free play** | 108.22 (48.62) | 2.40-213.23 | 36.59 | 126.19 (77.50) | 3.5-251.08 | 39.37 | 0.582 | |  |
| **Semi-structured play** | 286.73 (129.16) | 22.69-723-65 | 40.68 | 273.54 (133.85) | 86.65-697.28 | 36.44 | 0.403 | |  |

Supplementary Table 9b.

*Frequency of emotions “impossible to determine” in autistic and typical children in the entire MSPS and in each play period structure, as number of occurrences*

|  |  | |  |  | | | |  | | | | | |
| --- | --- | --- | --- | --- | --- | --- | --- | --- | --- | --- | --- | --- | --- |
|  | **Autistic (n=37)** | | | | | **Typical (n=39)** | | | | |  | |  |
|  | *M* (SD) | | Range | | Mean rank | *M* (SD) | Range | | Mean rank | *p* | |  |  |
| **Entire MSPS** | | 85.99 (29.51) | 17-121.11 | | 38.62 | 66.51 (24.45) | 11-121 | | 38.38 | 0.963 | |  |  |
| **Free play composite** | | 33.21 (11.41) | 8-57 | | 37.83 | 24.56 (9.8) | 8-47 | | 36.24 | 0.749 | |  |  |
| **Semi-free play** | | 17.76 (6.54) | 1-32 | | 39.26 | 13.79 (5.82) | 1-24 | | 36.78 | 0.621 | |  |  |
| **Semi-structured play** | | 35.02 (16.88) | 2-85 | | 38.47 | 28.16 (14.45) | 2-65 | | 37.51 | 0.849 | |  |  |
