# Supplementary figures and images for: Positive, negative, neutral—or unknown? The perceived valence of emotions expressed by young autistic children in a novel context suited to autism
Source: Autism. 2022 Feb 16;26(7):1833–48. doi: 10.1177/13623613211068221 (PMC9483191; doi:10.1177/13623613211068221)

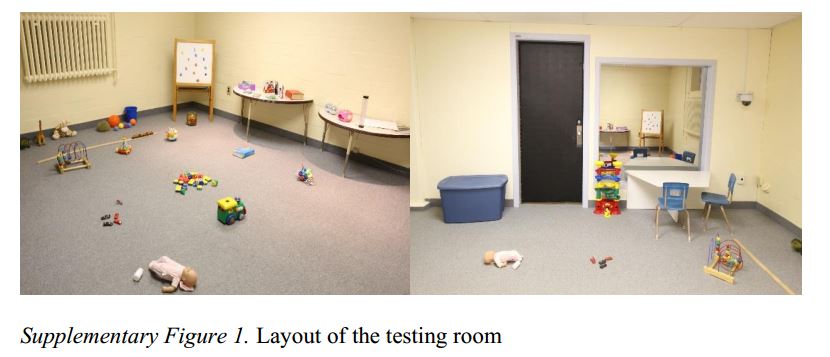

Supplement: sj-JPG-2-aut-10.1177_13623613211068221 – Supplemental material for Positive, negative, neutral—or unknown? The perceived valence of emotions expressed by young autistic children in a novel context suited to autism [file sj-JPG-2-aut-10.1177_13623613211068221.JPG]
